# Immunomodulation—a general review of the current state-of-the-art and new therapeutic strategies for targeting the immune system

**DOI:** 10.3389/fimmu.2023.1127704

**Published:** 2023-03-09

**Authors:** Martyna Strzelec, Jan Detka, Patrycja Mieszczak, Małgorzata Katarzyna Sobocińska, Marcin Majka

**Affiliations:** Department of Transplantation, Institute of Pediatrics, Faculty of Medicine, Jagiellonian University Medical College, Krakow, Poland

**Keywords:** immune system, immunomodulation, inflammation, RNA interference, mesenchymal stem cells, anti-inflammatory, autoimmune diseases, monoclonal antibodies

## Abstract

In recent years, there has been a tremendous development of biotechnological, pharmacological, and medical techniques which can be implemented in the functional modulation of the immune system components. Immunomodulation has attracted much attention because it offers direct applications in both basic research and clinical therapy. Modulation of a non-adequate, amplified immune response enables to attenuate the clinical course of a disease and restore homeostasis. The potential targets to modulate immunity are as multiple as the components of the immune system, thus creating various possibilities for intervention. However, immunomodulation faces new challenges to design safer and more efficacious therapeutic compounds. This review offers a cross-sectional picture of the currently used and newest pharmacological interventions, genomic editing, and tools for regenerative medicine involving immunomodulation. We reviewed currently available experimental and clinical evidence to prove the efficiency, safety, and feasibility of immunomodulation *in vitro* and *in vivo*. We also reviewed the advantages and limitations of the described techniques. Despite its limitations, immunomodulation is considered as therapy itself or as an adjunct with promising results and developing potential.

## 1 Introduction

The immune system has an invaluable role in the resistance to pathogenic infections and the maintenance of homeostasis. An adequate immune response to an encountered danger is an eligible and a deliberate balance-saving mechanism (1–4). However, an amplified and out-of-control immune response can act as a self-driving positive feedback loop and can have severe implications and is tightly connected to the development of a wide range of diseases (5, 6). Some of them have their source in the chronic inflammatory process, while others result from the buildup of an abnormal immune response against particular cells, thus leading to the development of autoimmune diseases. There is no doubt that the progression of inflammation conduces to disease aggravation and deterioration of patient status. It is legitimate to externally attenuate the immune response because an increasing number of evidence indicates that chronic inflammation, manifested, i.e., by an increase in the levels of proinflammatory cytokines, is involved in the pathogenesis of many diseases including asthma, rheumatoid arthritis, hepatitis, heart disease, and even some of the most prevalent disorders of the central nervous system (CNS) such as Alzheimer’s disease, epilepsy, depression, and schizophrenia. Immunomodulation is a potent branch, with a steady progress in pharmacokinetics and pharmacodynamics. There are many difficulties that need to be faced such as attenuation of the side effects in pharmacological treatment (7, 8) or toxicity in the newest interventions (9). However, so far, immunomodulation is successfully applied in the clinic (10, 11).

The immune system is a multiscale system that involves genes, molecules, cells, and organs, organized in complex networks of synergistic interactions and aimed at combating various types of threats to the organism (12). Our systematically expanding knowledge about this complex network has enabled us to more selectively influence its individual components, allowing a more effective treatment of many diseases. For example, in oncology, it is possible to enhance the natural ability of human T cells to recognize tumor cells (11, 13). The paracrine anti-inflammatory properties of mesenchymal stem cells (MSCs) are used in the treatment of some diseases (described further in a subsection in this article). Another possibility is to inactivate specific proinflammatory factors (i.e., TNF-α, IL-6) using monoclonal antibodies (14, 15). This clinical branch also benefits from novel methods of antibody design and production (16, 17). Monoclonal antibodies can be also used as agonists to imitate immunomodulatory signaling on antigen-presenting cells. We also can inhibit proinflammatory factors by blocking their release (18–20). There is also a wide range of pharmaceuticals modulating inflammasome functioning (21, 22). Also, well-established immunomodulatory therapies, such as corticosteroids, non-steroidal anti-inflammatory drugs, histamine antagonists, and interferons, have a new face in the context of research aiming to improve their efficiency. In brief, there are various potential targets in immunomodulatory intervention which efficiently modulate the immune response at its many stages. Immunomodulation, based on our knowledge about immunity, has not yet been fully explored but constitutes a perfect tool or an adjunct to regulate some disease progression.

In this review, we introduce the mechanisms of immunity and immunomodulation strategies used in basic science and in the clinic. Immunomodulation strategies are described cross-sectionally starting from well-established pharmaceutical interventions, through biological strategies, to the most recent scientific achievements as genome editing and regenerative medicine tools.

## 2 Innate immune response

Innate immunity is the first line of defense against pathogens. This kind of immune response is not selective and does not result in immune memory; however, it is rapid in reaction. It involves epithelial barriers and phagocytes, which are a group of myeloid cells, composed of neutrophils, dendritic cells, blood monocytes, and tissue macrophages. The innate immune response involves also the complement system, natural killer (NK) cells, and tissue-resident immune cells (23). An example of the regulation of this type of immunity is paracrine secretion of residual MSCs, which can modulate the functions of tissue macrophages, which is discussed further in this article.

In case of the presence of signals, which indicate infections or death of neighboring cells, phagocytes can intercept pathogen-associated molecular patterns (PAMPs) or damage/danger-associated molecular patterns (DAMPs) by pattern recognition receptors (PRRs) on their surface (24–26). The pathogen is internalized by the phagocyte and digested down into component proteins, which are afterward exposed to the cells of the adaptive immune system *via* major histocompatibility complex II (MHCII) on the surface of the phagocyte. Simultaneously, to multiply the immune response, at the initial moment of PRR activation, nuclear factor kappa-light-chain-enhancer of activated B cells (NF-κB) is activated (27, 28) and initiates the transcription of pro-IL-1β and pro-IL-18 and the inactive form of inflammasomes such as inflammasome NLRP3. This multimeric protein is involved in the activation of caspase-1 enzyme, which in turn converts proinflammatory cytokines to their active form. However, only in case of additional stimuli, which indicate a potential threat to homeostasis, i.e., potassium efflux, calcium influx, mitochondrial damage, or the presence of reactive oxygen species (ROS), cathepsins, or extracellular ATP, the dimerization of caspase-1 occurs, and pro-IL-1β and pro-IL-18 can be activated *via* proteolytic cleavage. Mature cytokines are secreted and activate the subsequent inflammatory pathways. A group of monoclonal antibodies is used as interleukin inhibitors in the clinic in diseases such as atopic dermatitis (dupilumab), plaque psoriasis (ustekinumab, ixekizumab), and COVID-19 infections (tocilizumab). In a phase 2 clinical trial, the IL-6 receptor inhibitor sarilumab was used in combination with other monoclonal antibodies in patients with melanoma (NCT05428007). Interestingly, the NLRP3-mediated inflammatory pathway is the potential target of the beneficial and anti-inflammatory effects of some antidepressants, i.e., tianeptine, venlafaxine, fluoxetine, or reboxetine (29). Fingolimod (FTY-720), a modulator of sphingosine-1-phosphate (S1P) receptor used in the therapy of multiple sclerosis, is known to block NLRP3 inflammasome assembly by downregulating NLRP3, apoptosis-associated speck-like protein (ASC), and caspase-1, thus reducing the levels of TNF-α, IL-6, and IL-1β and promoting microglia polarization into the M2 phenotype (22, 30). Aside from the activation of proinflammatory cytokines, caspase-1 is also responsible for cleaving pro-gasdermin D proteins. The products of this cleavage are involved in a specific cell death—pyroptosis (31, 32).

Cellular innate immunity is also composed of NK cells, which have the ability to release factors to induce cell apoptosis. They do not require activation by specific antigens and are able to respond immediately when exposed to a pathogen. Their action is inhibited by major histocompatibility complex I (MHCI) which is expressed on the surface of all nucleated cells of the body and protects them from destructive NK-cell action. During some viral infections or carcinogenesis, body cells could suppress MHCI expression which leads them to be recognized as non-self and to be eliminated by NK cells (33, 34). The action of NK cells can be modulated by IL-22 secreted by T lymphocytes which indirectly suppress the function of NK cells against cancer cells by modulating CD155 expression on the cancer cells’ surface (35).

The innate immune system is not specific and creates a first line of barrier against pathogens. However, in case of an escalated infection caused by a specific pathogen, a proper, more specific immune response mechanism needs to be activated. This is done *via* antigen presentation to the adaptive immune system by antigen-presenting cells (APCs), i.e., dendritic cells, B cells, and macrophages. Dendritic cells with digested pathogens travel *via* circulation to the lymph nodes and present their antigens to the T-cell receptor (TCR) of naive T helper cells (Th0) within MHCII complexes on their surfaces. To become fully activated, Th0 cells also require co-stimulation from APCs, in the form of B7 proteins (CD80 or CD86) expressed on the dendritic cell surface, which binds to the T cell CD28. This promotes Th0 cell differentiation either into Th1 cells, which promote cytotoxic T cells and cell-mediated immunity, or Th2 cells, which promote B cells and humoral immunity (36, 37).

Activation of the innate immune system promotes induction of adaptive immunity because cellular innate immune system components, such as dendritic cells and also cytokines, are able to stimulate the proliferation, differentiation, and survival of lymphocytes.

## 3 Adaptive immunity

Adaptive immunity involves mostly lymphocytes, namely, T cells and B cells. It provides long-lasting immunity with highly specific clonal responses to a large diversity of antigens. The adaptive immune response is self-limiting and quickly declines as the infection is eliminated as it generates immune memory and self-reactivity.

### 3.1 Cell-mediated immunity

Cell-mediated immunity is the term for a specific adaptive immune response activated by Th1 cells, which leads to the activation of APCs and a cytotoxic T-cell response. This immune response fights intracellular infections, including viruses (38), some bacteria, fungi (39), and protozoans (40).

**Figure 1:**
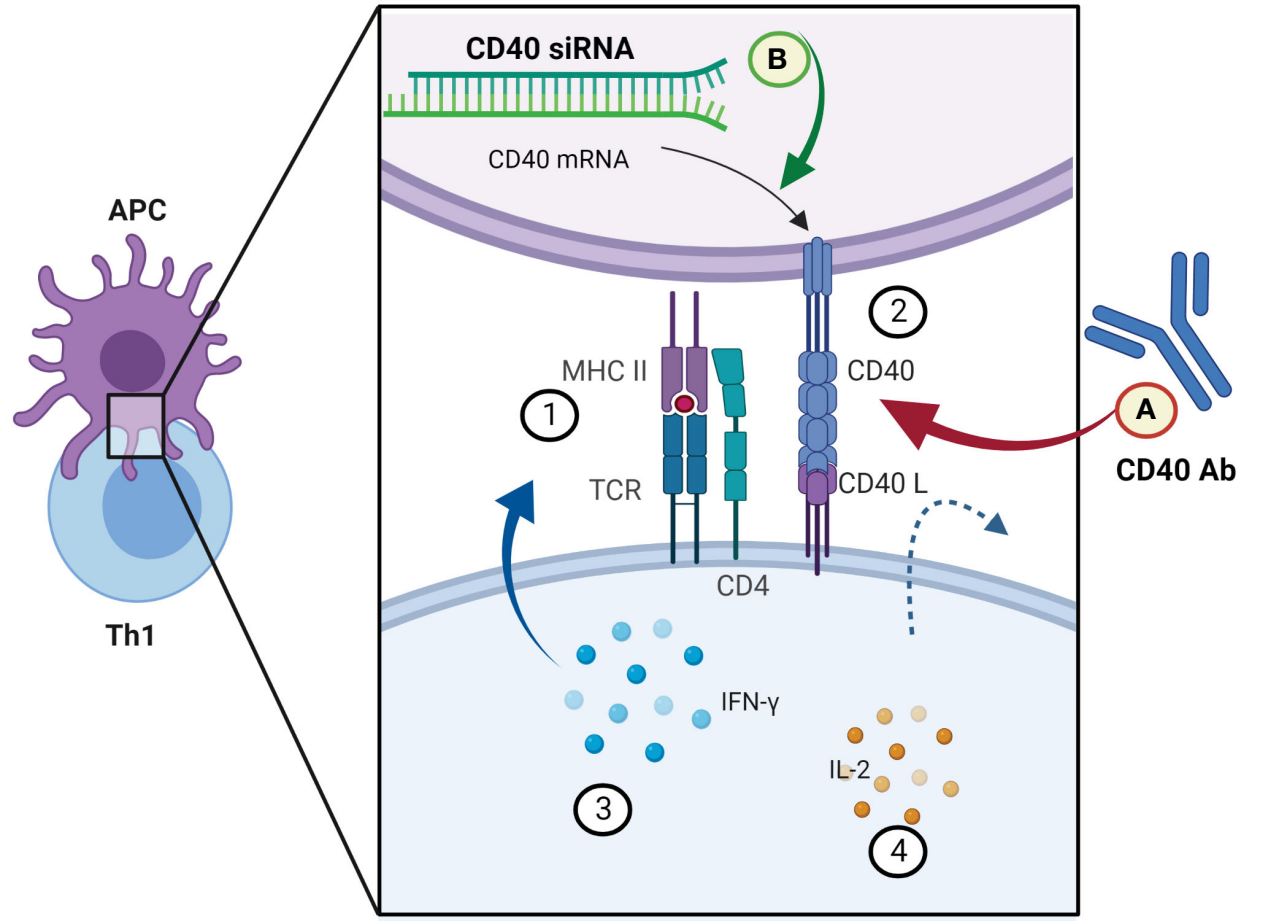
Examples of novel immunomodulatory strategies in modifying the interactions between CD40 and CD40L signal molecules. APC and Th1 signaling molecules in the first step of cytotoxic T-cell activation. (1) MHCII presenting antigen of the pathogen to the Th1 cell. (2) An additional signal in return activates the APC. (3) INF-γ intensifies the activation of the APC. (4) IL-2 released by Th1 cells stimulates cytotoxic T cells to enable the elimination of infected cells. **(A)** Co-stimulation of CD40L could be simulated by a monoclonal antibody (clinical trial nos. NCT03193190, NCT03424005, NCT03555149); **(B)** expression of CD40 in APCs (dendritic cells) can be downregulated by a specific siRNA (43). Created with BioRender.com.

APCs present pathogen epitopes using MHCII on their surface. Th1 cells recognize this signal by TCR and activate APCs by providing a second signal (CD40-CD40ligand) and release interferon-gamma (IFN-γ) (41). Activated APCs present antigen to the cytotoxic T cell within an MHCI along with a variety of second signals (B7+CD28 and/or 4-IBB+4-IBB ligand). This activation of cytotoxic T cells is enhanced with IL-2 released by Th1 cells (42). Once activated, the cytotoxic T cells identify infected cells by recognizing antigen displayed within MHCI on their surface. CD40 agonistic antibodies can imitate the arrangement of CD40L to CD40 and initiate immune antitumor signaling in macrophages, dendritic cells, and B cells and apoptotic signaling on tumor cells. Many studies evaluate the efficiency of CD40 agonistic antibodies alone or in combination with other treatments (NCT03193190, NCT03424005, NCT03555149) (**Figure 1**). On the other hand, also the inhibition of CD40-mediated effects can be a viable therapeutic strategy in the context of inflammatory diseases and prevention of allograft rejection. So far, in preclinical animal studies, attenuation of CD40 gene expression in dendritic cells with siRNA resulted in permanent acceptance of heart allografts in mice (43). Moreover, there are studies which evaluate analogs that prevent APCs from delivering the co-stimulatory signal in autoimmune and inflammatory diseases. In a phase 3 clinical trial (NCT05428488), the level of rheumatoid arthritis remission in the group treated with TNF-α inhibitors is a reference to the efficiency of treatment consisting of abatacept, which is a soluble cytotoxic T lymphocyte antigen 4 (CTLA-4).

Cytotoxic T cells remove infected cells in various ways. They perforate the cell wall of infected cells and release granzymes, granulysin, and perforins, which induce apoptosis and DNA fragmentation. They can also lead to forming a death-inducing signaling complex (DISC) by Fas ligand interactions (44). Cytotoxic T cells also release IFN-γ which blocks intracellular viral replication to prevent the spread of viral infection. Some of the specific cytotoxic T cells after infection become dormant memory T cells. Their role during reinfection is to speed up and enhance secondary pathogen recognition and elimination (45, 46).

### 3.2 Humoral response

Humoral immunity is the term for a specific adaptive immune response activated by Th2 cells which leads to the production of B cells and antibodies. This immune response fights with extracellular infections, including bacteria, fungi, protozoans, and parasitic infections. This immune response can also support intracellular infections (47, 48).

**Figure 2:**
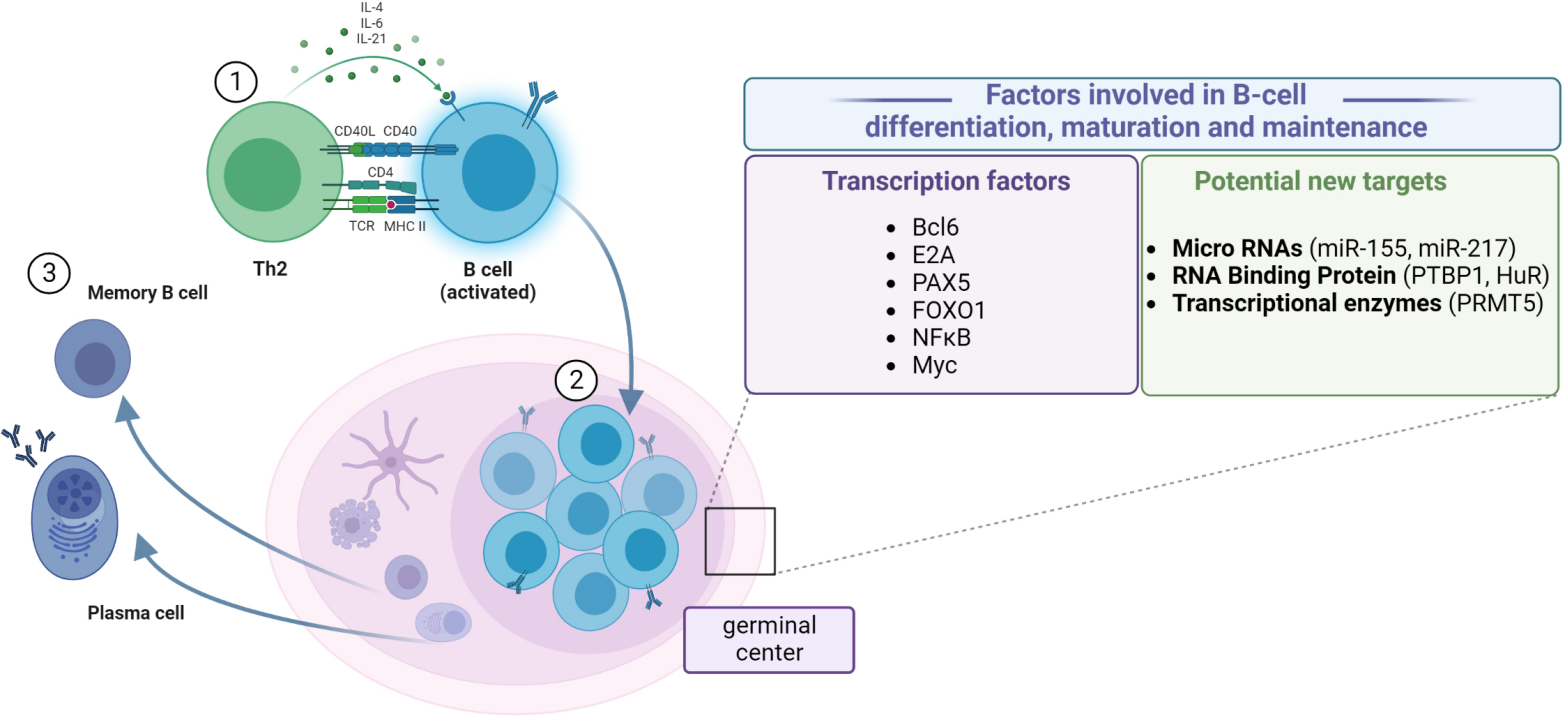
Steps of B-cell differentiation and maturation. (1) Antigen recognition induces the expression of effector molecules by T cells, which activate the B cells. (2) B-cell proliferation in the germinal center. (3) Differentiation between resting memory cells and antibody-secreting plasma cells. Establishing the role of particular factors in the regulation of transcriptional and post-transcriptional mechanisms of B-cell differentiation, maturation, and maintenance, which could help design better treatment and vaccination procedures. Created with BioRender.com.

Once naive Th0 cells have been activated by their specific antigen, they differentiate into Th2 cells. Th2 cells create a connection with B cells by the TCR–MHCII complex. B cells belong to APCs, which means that they can recognize pathogens and digest them and present their antigens on the surface by MHCII. The second signal is created by Th2’s CD40 ligand and B cell’s CD40. Th2 cells also release cytokines which promote B-cell development. Activated B cells mature into plasma cells (which produce antibodies) or dormant “memory” B cells (which are responsible for secondary pathogen recognition). Maturation and differentiation of B cells in germinal centers is a multistage and multifractional phenomenon, the mechanism of which is still under investigation. The well-established transcription factors involved in B-cell maturation, differentiation, and maintenance are as follows: Bcl (49); E2A, PAX5, and FOXO1 (50); NF-κB (51–53); and Myc (54, 55). Recently, microRNAs (56, 57), RNA-binding proteins (58), and transcriptional enzymes (59) have attracted much attention as potential targets to regulate B-cell functioning (60) (**Figure 2**).

Antibodies can neutralize pathogens in a number of ways: 1) they can directly bind to toxins and neutralize them; 2) they also can bind to antigens on pathogen surfaces; 3) they agglutinate pathogens to impair their mobility; and 4) they opsonize pathogens to enhance their phagocytosis. Binding to antigens activates the classical complement pathway. They can also activate effector cells such as dendritic cells, NK cells, and cytotoxic T cells (61, 62).

## 4 Inflammatory response

**Figure 3:**
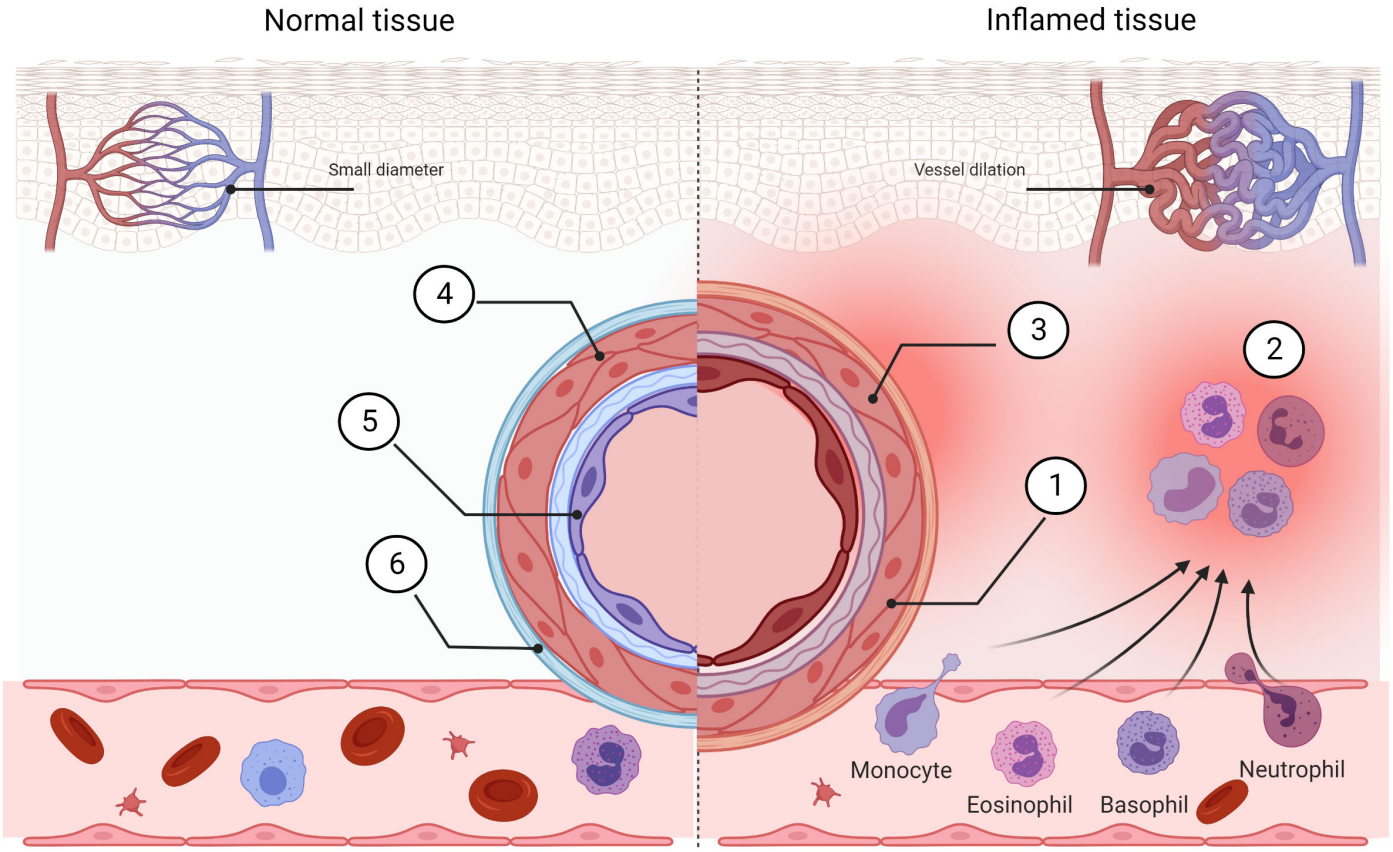
Comparison of normal tissue and inflamed tissue. Prostaglandins, bradykinin, histamine, and NO cause vasodilation in inflamed tissue. Blood flow is increased (1) and this allows morphotic particles of the blood to intensify streaming (2). Locally, the pain sensitivity is increased (3). The vessel in the normal stage exhibits low permeability (4) and normal phenotype of endothelial cells (5). This helps to inhibit immune cell extravasation (6). Created with BioRender.com.

The development and progression of the inflammatory process is tightly connected with the innate immune system action. The main initial features of inflammation include vasodilation and increased blood flow. This leads to erythema and an increase in the temperature of the inflammation-affected area. Increased vascular permeability allows the inflammatory cells to infiltrate from the blood flow to the tissue, causing tissue edema and swelling. Inflammatory mediators such as bradykinins and prostaglandins increase pain sensitivity and cause hyperalgesia (63). Cleaning up of the infected area is possible because of the chemotaxis ability of neutrophils triggered by a gradient of chemokines released by the damaged tissue (64) (**Figure 3**). Fever and “flu-like” symptoms like hot flushes, sweats, chills, rigors, headache, and fatigue force an infected organism to save energy to fight with the pathogen and are induced by an increase in inflammatory markers like CRP and ferritin. During an innate inflammatory response, upregulation of co-stimulatory molecules such as MHCII and B7 occurs to encourage activation of the adaptive immune system. An overactive or chronic inflammatory response lies at the core of numerous pathological conditions, and thus, many of the immunomodulatory drugs used today are aimed specifically at suppressing inflammation.

## 5 Classic pharmacological approach for immunomodulation

Immunosuppressants can modulate multiple sites of the immune response, starting with the influence on the transcription rate of genes encoding proteins necessary for lymphocyte action and then with the regulation of the final stages of the humoral response, e.g., regulation of the antibody titer and the degree of their affinity. They are applied in different clinical branches, including the treatment of autoimmune diseases and transplantations, using, i.e., corticosteroids, non-steroidal anti-inflammatory drugs (NSAIDs), histamine antagonists (HAs), and also a wide range of cellular signaling inhibitors.

### 5.1 Corticosteroids

Corticosteroids are stress hormones, which regulate cellular development, proliferation, metabolism, and immune response *via* binding to mineralocorticoid (MR) and glucocorticoid (GR) receptors. Due to their potent anti-inflammatory and immunosuppressive action, synthetic corticosteroids like dexamethasone, prednisolone, and budesonide are used in the treatment of respiratory diseases, such as asthma, chronic obstructive pulmonary disease (COPD), and acute respiratory disease (ARD), and also allergies and some autoimmune diseases, i.e., arthritis and lupus. Corticosteroids can be applied in many different forms, which include oral, intravenous, intramuscular, transdermal, and transmucosal or inhalation routes and may be also used as an adjuvant in combination treatment with other drugs (65). Currently, high-dose corticosteroids are also considered to treat severe inflammation associated with respiratory viral infections, such as influenza, COVID-19, or respiratory syncytial virus (66). However, it should be taken into account that corticosteroids, acting on all cell types, have a very complex and pleiotropic effect and, thus, produce many side effects. The advantages of corticosteroid therapies are as follows: effectiveness in a wide range of diseases, short time to obtain treatment effects, and multiple ways of administration and formulations. The disadvantages of corticosteroid treatment are as follows: they are not self-sufficient and need to be used in combination with other drugs. The burdensome side effects of prolonged corticosteroid treatment include altered response to physical stress, high blood pressure, weight gain, diabetes, loss of bone density, and an increased risk of infections (67). A new wave of corticosteroids is tested in clinical trials, e.g., vamorolone (VBP15) (NCT02415439, NCT05185622, NCT05166109). The main challenges in designing new corticosteroid therapies are to improve the risk/benefit ratio, increase tolerability in terms of both systemic and local adverse effects, and reduce the risk of sensitization (68).

### 5.2 Non-steroidal anti-inflammatory drugs

The use of the very first NSAID—aspirin—dates back to the 18th–19th centuries, when the antipyretic usage of willow bark gave rise to the isolation of salicin and finally to the synthesis of acetylsalicylic acid. The anti-inflammatory action of NSAIDs is based on the reduction of prostaglandin E2 and prostacyclin levels, preventing from local vasodilation. Additionally, other inflammatory mediators, such as histamines, cannot profusely stream into the capillaries; thus, they cannot intensify local vasodilation, which is a non-direct influence of NSAIDs on blood vessels. What is important, the cumulation of proinflammatory cells is not directly inhibited. Examples of NSAIDs are aspirin, ibuprofen, naproxen, indomethacin, piroxicam, and paracetamol. The inhibition of cyclooxygenase enzymes leads to prostaglandin synthesis inhibition (69). The anti-inflammatory, antipyretic, and analgesic effects are caused by the inhibition of isoform 2-cyclooxygenase (COX-2), and side effects connected with the intake of NSAIDs arise from the inhibition of the constitutive isoform 1-cyclooxygenase (COX-1) (70, 71). One strategy to decrease the side effects of NSAIDs is creating drugs of convectional structure with combined –NO and –H2S groups. Those drugs after hydrolysis in the plasma and intercellular lymph release NO which decreases ulceration (7). This strategy could also be used with coxibs (8). The coxibs such as celecoxib and etoricoxib exhibit higher selectiveness in the inhibition of COX-2; thus, they induce fewer gastrointestinal side effects. To sum up, the pros of NSAIDs are their anti-inflammatory, antipyretic, and analgesic actions and their antineoplastic, antithrombotic, and antiarthritic effects. The cons of NSAIDs are gastrointestinal complications, hepatotoxic problems, renal injury, cardiovascular problems, cerebral complications, respiratory tract issues, and mitochondrial toxicity (72). Future perspectives in designing new NSAIDs are to decrease gastrointestinal complications by adding –NO and –H2S groups or donors, like EV-34 (73), ATB-352 (74), and ATB-346 (NCT03291418, NCT03978208, NCT03220633) (75, 76). Another challenge in designing new NSAIDs is to find highly selective inhibitors of the COX-2 enzyme to increase the benefit/risk profile (77–80).

### 5.3 Histamine antagonists

Histamine has multiple effects by binding to its four G-protein-coupled histamine receptors. Due to their pleiotropic expression, the action of their antagonists can induce general side effects. The family of histamine antagonist drugs is composed of antagonists with four histamine receptors: H1, H2, H3 (81), and the most recently described H4 (82). However, the term antihistaminergic drugs relates primarily to antagonists of the H1 receptor. In the short term, H1 receptors are mostly involved in allergic inflammation, H2 receptors are involved in the regulation of gastric acid secretion, H3 receptors control neurotransmission, and H4 receptors are responsible for immunomodulation (83). The disadvantage of the first generation of histaminergic receptor antagonists is crossing the blood–brain barrier (BBB); therefore, they exhibit sedative properties. Contrary to the first generation, the second generation of antihistaminergic drugs cannot cross the BBB; thus, they do not exhibit sedative properties. Another advantage of the second generation of antihistamines is the fact that they do not exhibit anticholinergic side effects and do not impair psychomotor performance. Examples of new histaminergic receptor antagonists whose safety and efficacy were proven in clinical trials are JNJ39758979 (rheumatoid arthritis, asthma, histamine-induced itch, dermatitis), ZPL3893787 (atopic dermatitis, psoriasis), and UR-63325 (seasonal allergic rhinitis).

### 5.4 Cellular signaling inhibitors

Immunity is composed of multiple, interrelated pathways. Immune-related pathways could be modified, silenced, or switched off by cellular signaling inhibitors, such as Janus kinase (JAK) inhibitors (84, 85), calcineurin inhibitors (86–89), mTOR inhibitors (90), inosine 5′-monophosphate dehydrogenase (IMPDH) inhibitors (91), TACE (TNF-α-converting enzyme) inhibitors (19, 92), rho-associated protein kinase (ROCK) inhibitors (93), or interleukin-1 receptor-activated kinase 4 (IRAK4) (94) (**Table 1**).

**Table 1:** Characteristics of cellular signaling inhibitors.

Type of cellular signaling inhibitors	Characteristic	Example
JAK inhibitors	• Suppress the release of proinflammatory cytokines from the cell (85). • Approved for rheumatoid arthritis therapy. Small‐molecule JAK inhibitors are a novel category of drugs tested in clinical trials for immune‐mediated diseases and cancer (84).	Tofacitinib
Calcineurin inhibitors	• Act *via* suppressing T-cell activation (86, 87). • Used as an adjuvant to drugs for the prophylaxis of allogeneic post-transplant organ rejection (89) or after allogeneic organ grafts (88). • Calcineurin inhibitors are used for treating chronic atopic dermatitis (95).	Cyclosporine Tacrolimus
mTOR inhibitors	• mTOR regulates cell growth and proliferation, also associated with immune cell differentiation in immune regulation (96). • Show high antitumor activity in clinical studies. They are also used in combination with other antitumor drugs with a significant effect (90).	Sirolimus Everolimus
IMPDH inhibitors	• IMPDH is involved in purine metabolism and cell proliferation (97). Its activity is intensified in tumorigenesis (98). • Approved for a wide range of clinical uses such as the prevention of organ transplant rejection and antiviral agents and other indications including cancer and pathogenic microorganisms (91).	Azathioprine Leflunomide Mycophenolate
TACE inhibitors	• TACE is an enzyme that converts TNF-α and releases TNF-α related to the membrane (99, 100). • TACE inhibitors are promising and used in the clinic in rheumatoid arthritis, sepsis, other inflammatory disorders, and many aspects of cancer therapy (18, 19, 92).	TAPI-1 TMI-1, TMI-2, TMI-005 BMS-561392, BMS-566394
ROCK kinase inhibitors	• ROCK kinase regulates the shape and movement of cells by influencing the cytoskeleton (101). • Fasudil inhibits the expression of TLR4, MyD88, and NF-κB, which are key mediators of inflammation. The research showed that kinase ROCK inhibitors could suppress the activation of microglia and could shift astrocytes from an A1 to an A2 phenotype (10, 102, 103). • To increase the activity of Fasudil, researchers are working on its liposomal form (93).	Fasudil
IRAK4 inhibitors	• IRAK4 is an important mediator of inflammatory response, involved in the activation of the TLR and interleukin-1 receptor signal transduction pathways by binding to myeloid differentiation factor 88 (MyD88). • PF-06650833 reduced the levels of inflammatory markers in rodent models of systemic lupus erythematosus and rheumatoid arthritis in phase I clinical studies (94).	PF-06650833

## 6 Biologics

Current biologic strategies to modulate the action of the immune system rely on the application of either the naturally occurring or modified components of the innate and adaptive immune response, like interferons or monoclonal antibodies.

### 6.1 Monoclonal antibodies

The development of monoclonal antibodies (mAbs)—laboratory-engineered immunoglobulins directed against a specific epitope of a selected antigen—brought up a considerable potential for the treatment of cancer and also autoimmune diseases due to their highly selective binding capacity. The first method for the large-scale production of mAbs was developed by Georges Köhler and César Milstein in 1975 and involved the fusion of mouse myeloma cells with spleen cells from immunized animals (106). Monoclonal antibodies produced with this method (suffix: -omab) have rather low clinical efficacy due to their short half-life and high immunogenicity due to their murine origin. Thus, many improvements in the production of mAbs have been developed, including the introduction of chimeric (suffix: -iximab), humanized (suffix: -zumab), and fully human monoclonal antibodies (suffix: -umab). Up to date, approximately 18 monoclonal antibodies have been approved for the treatment of autoimmune diseases, such as rheumatoid and psoriatic arthritis, ankylosing spondylitis, ulcerative colitis, plaque psoriasis, and Crohn’s disease (107). The molecular targets include mostly proinflammatory cytokines, their receptors, or adhesion molecules present on the surface of particular immune cell types. Some notable examples of mAbs widely used in the clinic include golimumab (anti-TNF-α), ofatumumab (anti-CD20), ocrelizumab (anti-CD20), infliximab (anti-TNF-α), tocilizumab (anti-IL-6) and belimumab, which binds to soluble B lymphocyte stimulator of B cells (BLyS) (14, 15, 108). Most recently, anifrolumab, which is a fully human immunoglobulin gamma 1 kappa (IgG1κ) mAb raised against type 1 interferon receptor (IFNAR1), has gained approval in the United States for the treatment of systemic lupus erythematosus (109).

The development of new technologies for mAb production and selection, such as phage display, single B-cell isolation, or the use of transgenic fully human or chimeric human antibody mouse strains, enabled to obtain more refined therapeutic end products in contrast to the classical mouse hybridoma technique (110). However, it is worth emphasizing that many of mAb-based therapies for autoimmune disease often result in immunogenicity and the development of a high percentage of antidrug antibodies (ADAs), which although rarely produce adverse effects in patients, they can significantly weaken the therapeutic action of mAbs (111). Dual-affinity retargeting (DART) is a new antibody production process providing biospecificity, thereby facing the problems associated with conventional monoclonal antibodies (16, 17). The first dual-affinity recombinant protein registered as an immunomodulatory drug is Telitacicept, targeted against BlyS, and a proliferation-inducing ligand (APRIL), which was registered for the treatment of systemic lupus erythematosus in China (112).

### 6.2 Interferons

Interferons are a family of mammalian cytokines, which are secreted from the cells of the host organism in response to infection, but they are also involved in cell growth and immunomodulation. Depending on their structural homology, mode of action, and receptor preference, IFNs can be classified into three major types. In humans, type I interferons consist of IFN-α, IFN-β, IFN-ϵ, IFN-κ, and IFN-ω; type II is represented only by IFN-γ; and finally, the most recently described type III includes four subtypes of IFN-λ (113, 114). Among all endogenous cytokines involved in the regulation of immune response, up to date, interferons proved to be one of the most therapeutically useful in targeting a significant array of different diseases. IFN-γ is secreted mostly by T cells in response to viral and non-viral pathogens (bacteria and their secretome, *Rickettsia*, parasites, fungal polysaccharides, and cytokines). IFN-α and IFN-β are secreted by T and B cells, macrophages, and fibroblasts in response to viruses and cytokines. Interferons block viral replication *via* the induction of enzyme synthesis which inhibits the translation of viral mRNA. Interferons have a wide range of actions and inhibit the replication of most viruses *in vitro*. In the clinic, IFN-α is applied in chronic viral hepatitis types B and C. This interferon exhibits inhibition of herpes zoster and is used in common flu prevention. Its antitumorigenic (in lymphoma and solid tumors) action is still being evaluated. IFN-γ combined with antibacterial drugs is used in the treatment of chronic granulomatous diseases (113).

IFN-β is perhaps the most clinically useful, as both IFN-β-1a and IFN-β-1b are applied in the therapy of multiple sclerosis. Their mechanism of action is complex and not fully elucidated: it includes mitigation of the progression of inflammatory processes *via* regulation of the balance between pro- and anti-inflammatory cytokine release, inhibition of T-cell activation, and their migration across the BBB, as well as it was also shown to improve the proliferation and differentiation of neural stem cells (NSCs) in *in-vitro* conditions (113, 115). Up to date, several formulations of INF-β are being employed for multiple sclerosis (MS) treatment, including intramuscular IFN-β-1a (Avonex, Biogen), subcutaneous IFN-β-1a (Rebif, EMD Serono), and PEGylated IFN-β-1a (Plegridy, Biogen). Several long-term double-blinded, placebo-controlled studies conducted so far with different IFN-β-1a or IFN-β-1b formulations have shown their effectiveness in slowing/attenuating the progression and alleviating MS symptoms in patients suffering from various forms of this disease. The same studies, however, also indicate that IFN-β is the most effective when initiated in the early onset of MS symptoms (116). Although IFN-based therapies produce only partial long-term responsiveness when compared with the newly developed immunomodulatory drugs, they are generally well-tolerated by patients, and therefore, they still remain an important class of MS therapeutics.

## 7 Genomic strategies in immunomodulation

### 7.1 CRISPR–Cas9

**Figure 4:**
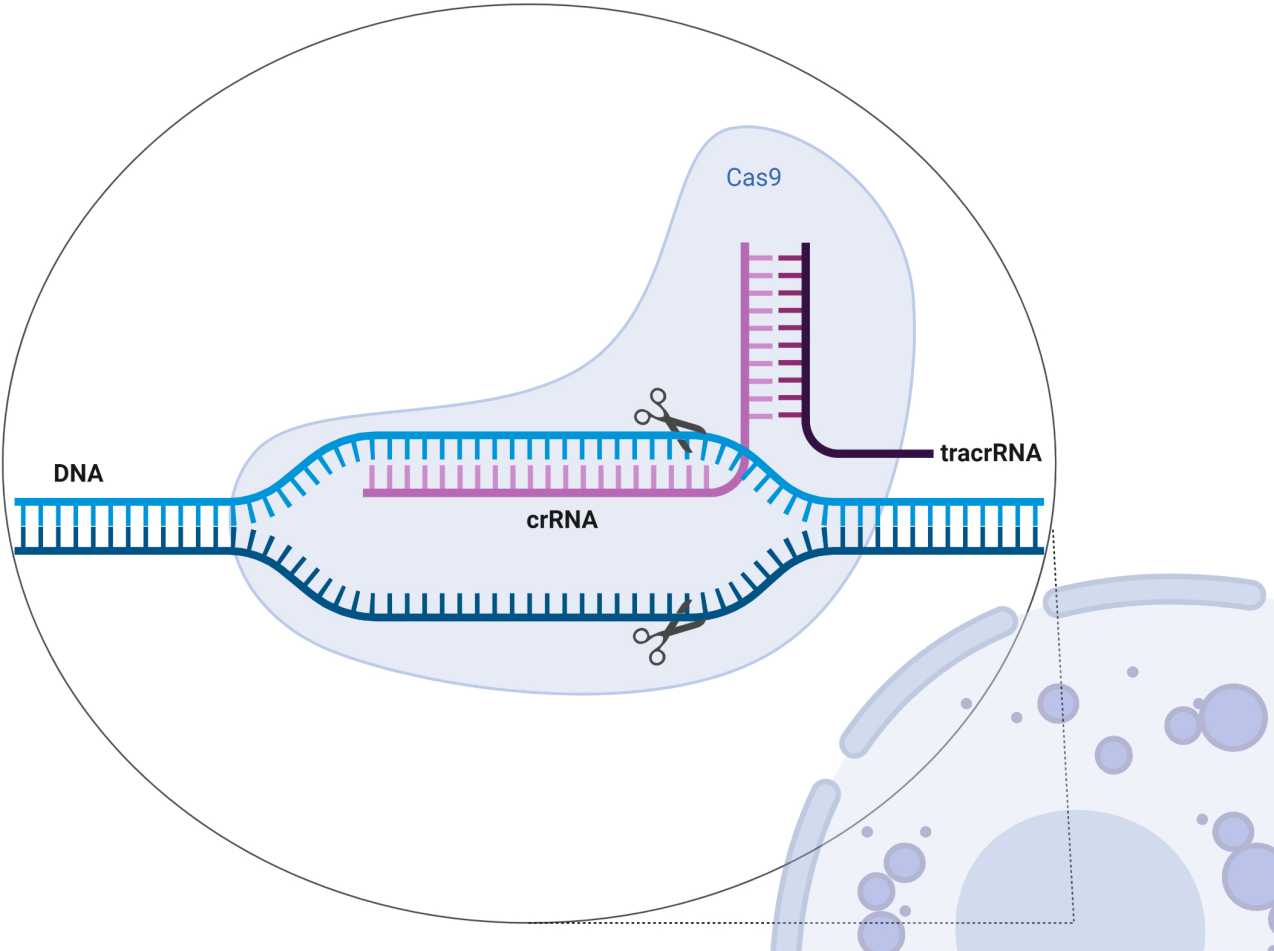
Scheme of the CRISPR/Cas9 structure. The CRISPR–Cas9 system is composed of two elements: crRNA–tracrRNA chimera and Cas9 (121). Created with BioRender.com.

CRISPR–Cas9 (clustered regularly interspaced short palindromic repeats–CRISPR-associated protein 9) genome editing is based on the CRISPR–Cas9 machinery of bacterial “adoptive immunity” (117–119). CRISPR-clustered repeats in *Escherichia coli* were reported for the first time in 1987 (120, 121) and later recognized in 2000 in prokaryotes (117) and formed the foundation of a groundbreaking technique in genome editing (122, 123) (**Figure 4**). The technique works as molecular scissors and enables editing of the chosen genes by their inactivation (cut-out of the gene by non-homologous end joining, NHEJ) or replacement (cut-out and replacement by homologous end joining, HEJ) (124, 125). There are many applications of this technique. It has been used for the detection of specific DNA targets by CRISPR screening for focused gene discovery (126–129). The group of Dr. Howard E. Gendelman (by applying CRISPR–Cas9) has proven for the first time that viral eradication is possible in an animal model (130). It has been shown that the CRISPR/Cas9 system can be used as a novel technology for the investigation of the pathogenesis and treatment of viral infections such as human immunodeficiency virus infection (HIV), hepatitis virus infections (HBV), immunological diseases, and also autoimmune diseases (131). The CRISPR/Cas9 technology is emerging as the preferred approach for gene editing, with its ease of use and nearly limitless DNA sequences that can be targeted (132).

So far, the first human clinical trial with CRISPR–Cas9 was performed to treat β-thalassemia (NCT03655678) and sickle cell disease (NCT03745287). It involved targeting *BCL11A* erythroid-specific enhancer in CD34^+^ hematopoietic stem and progenitor cells (133). CRISPR–Cas9 was used in gene editing of T cells to enhance the natural ability of these cells to fight refractory cancers such as multiple myeloma, cell liposarcoma, and non-small cell lung cancer (11, 13).

Apart from potential therapeutic applications in the future, CRISPR–Cas9 may prove an extremally useful tool in basic research, aimed at identifying key genes involved in the pathogenesis of autoimmune diseases, thus allowing for a better understanding of the mechanisms involved in autoimmunity. For example, deletion of the gene in the RAW264.7 macrophage cell line using the CRISPR/Cas9 system designed to target the miR-155 gene, associated with rheumatoid arthritis, resulted in the reduced expression of proinflammatory cytokines (134). It was also found that deletion of the rs6651252 enhancer in the HCT116 cell line regulates the expression of the c-MYC proto-oncogene (MYC), associated with Crohn’s disease and ulcerative colitis (135). In recent years, CRISPR–Cas9 genome editing also has contributed to the identification of cxorf21 as an important factor associated with the pathogenesis of systemic lupus erythematosus (136).

CRISPR–Cas9 is a very convenient tool for the precise editing of genes of interest and is undoubtedly one of the most promising technologies to be utilized in the clinic in the future. For now, however, CRISPR–Cas9 still mainly remains a technique implemented in preclinical research, performed mostly in *in-vitro* conditions. There are several important limitations, which prevent its use as a safe and viable therapeutic strategy, which mostly include effective delivery systems, as well as it raises concern about the consequences of its off-target actions (137). Other than CRISPR–Cas9, different CRISPR–Cas machineries are being used for nucleic acid editing or detection, e.g., CRISPR–Cas12 (138) and CRISPR–Cas13 (139).

### 7.2 RNA interference

Similar to the CRISPR–Cas9 technique, artificial RNA interference (RNAi) has its origin in naturally occurring immunity. RNAi mediates resistance to both endogenous parasitic and exogenous pathogenic nucleic acids and regulates the expression of protein-encoding genes in living organisms. RNAi is a biological process in which RNA molecules—microRNA (miRNA) and small interfering RNA (siRNA)—inhibit gene expression, typically by binding to messenger RNA (mRNA) and triggering its degradation. The most notable difference between the two is that miRNAs can bind to multiple targets and siRNAs require full complementary for binding to specific mRNA (140, 141). The process is exploited by researchers to screen for gene function and knock down gene expression in cell cultures and in organisms (142–144). In 2018, the US Food and Drug Administration approved patisiran (Onpattro), the first siRNA drug for the treatment of polyneuropathies induced by hereditary transthyretin amyloidosis (145), and in the following years, two other drugs, givosiran and lumasiran, gained approval, respectively, for the treatment of acute hepatic porphyria and type 1 primary hyperoxaluria (146, 147). There is a wide range of immune diseases in which the efficiency of RNAi treatment is tested in preclinical trials, e.g., HIV infection/AIDS, lymphoma, chronic hepatitis B, advanced solid tumors, and relapsed or refractory B-cell lymphoma (148).

The successful delivery of RNA molecule to the target cell is however not an easy task, not only because of physical properties preventing its passage through the lipid bilayer but also due to endogenous nucleases and macrophages and difficulties in crossing the extracellular matrix and cell membrane by receptor-mediated endocytosis. Therefore, numerous modifications of RNA molecules (including 5′- and 3′-end conjugates, 2′-sugar substitution, and internucleoside linkage) and delivery strategies, such as lipid nanoparticles, cationic polymers or exosomes, spherical nucleic acids, and DNA nanostructures, are being developed to improve RNA stability and absorption (148, 149).

Moreover, perhaps the most important thing to consider in the context of immune-based disease treatment is the development of an efficient way for the selective introduction of the desired RNA molecule into a particular type of immune cells. For example, in a recent work, myeloid cell-selective inhibition of NF-κB was achieved using a mimic oligonucleotide of miR146a conjugated to CpG motif to act as an agonist for Toll-like receptor 9 (TLR9), which allowed to knock down inflammatory and tumorigenic NF-κB activity in macrophages and myeloid leukemia in both *in-vitro* and *in-vivo* conditions (150). In another study, 1,3-β-glucan molecule (schizophyllan, SPG) was attached to poly-dA extension at the 5′-end of the siRNA sense strand, which allowed its selective incorporation into dendritic cells (DCs) through Dectin-1, which enabled the silencing of the CD40 gene (43). Some advances have also been made in the efficient delivery of miRNAs to the brain, for example, to effectively treat the effects of autoimmune CNS diseases such as MS. In one study, extracellular vesicles (EVs) with overexpression of miR-219a-5p stimulated oligodendrocyte precursor cell differentiation into mature, myelin-producing oligodendrocytes in an experimental autoimmune encephalomyelitis (EAE) model of MS, and furthermore, they were proven to be much more effective than liposomes and polymeric nanoparticles in crossing the blood–brain barrier (151). This research strongly suggests that miR-219a-5p can be effective in promoting remyelination in MS patients and also strongly supports EV as a viable method for miRNA delivery into the CNS.

Taken together, research into different therapeutic approaches based on RNAi mechanisms may bring invaluable benefits in the treatment of inflammatory and autoimmune diseases. The introduction of new siRNA and miRNA-based drugs requires, however, the precise identification of specific therapeutic targets and the development of effective methods for the selective introduction of RNA molecules into particular cells and organs in order to prevent its off-target activity.

## 8 Immunomodulatory properties of mesenchymal stem cell therapies

MSCs were discovered in 1970 (152), and they are multipotent adult cells with self-renewing properties. These cells have immunomodulatory features; therefore, MSCs are potential tools in treating inflammation-related diseases. The common sources of MSCs are the bone marrow (BMMSCs), adipose tissue (ATMSCs), and perinatal tissues, such as Wharton’s jelly (WJMSCs) and amniotic fluid (AFMSCs). The features of MSCs are high, multilineage proliferative potential; low immunogenicity; specific migration to the sites of tissue injury; and immunomodulatory potential. Paracrine secretion triggers anti-apoptotic activity, angiogenesis, and anti-fibrosis and reverses remodeling (153).

The Mesenchymal and Tissue Stem Cell Committee of the International Society for Cellular Therapy determines that MSCs could be differentiated by their phenotypic features, like the expression of CD105, CD73, and CD90 and the lack of CD34, CD45, CD14 or CD11b, CD79a or CD19, and HLA-DR expression. MSCs could be also separated by their morphological properties, i.e., adherent, fibroblast-like morphology in standard culture conditions, and they are able to cluster into fibroblast colonies, potent to multidirectional differentiation at least into osteoblasts, adipocytes, and chondrocytes (154). The biological role of MSCs is supportive: derivates of MSCs (osteoblasts and fibroblasts) co-create marrow niches for hematopoietic cells. Stromal cells take part in hematopoietic regulation; they secrete growth factors, chemokines, and cytokines (GM-CSF, LIF, SCF, thrombopoietin, IL-8, IL-10, IL-11, IL-14, IL-15). MSCs are involved in tissue regeneration and immunomodulation. They enable the settlement of the marrow environment by transplanted hematopoietic stem cells.

In a low concentration of environmental INF-γ or TNF-α, MSCs are polarized into the M1 phenotype. They express a high level of TLR4 to which LPS can be ligated. Then, MSCs M1 secrete low levels of IDO, NO, and PGE2 but high levels of CXCL9, CXCL10, MIP-1a/β, and Regulated upon Activation, Normal T Cell Expressed and Secreted (RANTES) (155, 156). This activates T0 cells to become T cytotoxic cells with a high expression of CCR5 and CXCR3. Within the negative feedback loop, activated T cytotoxic cell secretes a high level of IFN-γ and TNF-α to initiate an anti-inflammatory pathway of MSCs. Collaterally, MSCs M1 stimulate monocytes into the M1 phenotype (CD86 positive) (157), which secretes a high level of IFN-γ and TNF-α to the environment.

MSCs exhibit infinitesimal immunogenicity. They have a low expression of HLA-I, which increases after exposition to INF-γ, and they also have a low expression of HLA-II. MSCs exhibit a lack of antigen-specific response because of the lack of the co-stimulant molecules CD80 and CD86. MSCs express non-specific HLA-I antigens, which are involved in the tolerogenic process occurring in the fetal–maternal interface: HLA-G, HLA-G5 (induction of regulator T cells and suppression of INF-γ production from natural killer cells), HLA-E, and HLA-F (158). WJMSCs exhibit a higher expression of HLA-G antigens in comparison to other types of MSCs. MSCs have a high and multidirectional proliferation potential *in vitro*. They spontaneously migrate to the area of injury (159). MSCs produce components of the extracellular matrix: collagen types I, III, IV, and VI; fibronectin; laminin; hyaluronan; and proteoglycans. They suppress the proliferation of alloT lymphocytes after transplantation. There are no antibody anti-surface proteins of MSCs. In the presence of MSCs, mitogen-stimulated lymphocytes do not proliferate. Transplantation of MSCs does not cause tolerance induction for HLA antigens of given stem cells (160, 161).

MSCs have a wide range of impacts on T cells. They inhibit the proliferation of T cells in response to mitogens, anti-CD3, anti-CD28, or alloantigens. MSCs induce anergy of naive T cells, induce T regulator cell expansion, and inhibit the expression of CD25 and CD69 (inhibition of activation). They also inhibit the alloreactivation of cytotoxic T cells. The immunomodulatory action of MSCs is conditioned by the environment of the cells. In a high concentration of environmental INF-γ or TNF-α, MSCs are polarized into the M2 phenotype. They express a high level of TLR3 to which dsRNA can be ligated (162). Then, MSCs M2 secrete high levels of IDO, NO, PGE2, HGF, and TGF-β. TGF-β, PGE2, and sHLA-G factors evoke T0 cells to become T regulator cells (CD4, CD25, FoxP3 positive). Additionally, IDO and PGE2 secreted by MSCs M2 stimulate monocytes into the M2 phenotype (CD206 and CD163 positive), which in turn secrete anti-inflammatory cytokines (Il-6, IL-10, and CCL-18) (157).

MSCs are applied in clinical settings. By May 2022, there were 1,022 registered clinical trials worldwide involving over 10,000 patients investigating the therapeutic potential of MSCs. Their differentiation into osteoblasts is used in osteonecrosis and osteogenesis imperfecta treatment. MSCs can be used in the reconstruction of the cartilage, e.g., nose, ears, or trachea. MSCs after transplantation accelerate hematopoietic reconstitution in hematopoietic cancers. They also accelerate hematopoietic reconstitution in non-hematopoietic cancers, like breast cancer. MSCs are widely used in orthopedics: implantation of an endoprosthesis along with MSCs and hydroxyapatite accelerates bone regeneration.

The trophic factors secreted by MSCs have beneficial effects on the central nervous system, thus making MSC-based therapies suitable candidates for the treatment of CNS injuries and neurodegenerative diseases. In light of the current scientific data, the MSC secretome was shown to display both direct and indirect influences on neuronal and glial survival and differentiation (163), peripheral nerve regeneration (164), and potency to induce neurite growth (165). On one hand, the MSC exosomes possess immunomodulatory properties and can have an impact on M2-type macrophages in the injured spinal cord (166).

In lower limb ischemia, MSC transplantation can result in the improvement of vascularity (167). MSCs when used on hard-to-heal wounds can result in skin reconstruction and closing of the wound.

MSCs are also used to treat cardiomyopathy or myocardial infarction. The mechanism of action of MSCs in cardiovascular diseases is proposed to be direct cell–cell contact (respiratory chain salvage and stimulation of differentiation). They directly differentiate into cardiomyocytes and smooth muscle cells. It is speculated that they can also differentiate into endothelial cells. The efficacy of MSCs in cardiac diseases was proven by many preclinical and clinical trials (168–176).

MSCs also inhibit inflammation by anti-graft-versus-host disease action (153, 177). They could be widely used in the treatment of steroid-resistant acute graft-versus-host disease (GvHD) by their immunosuppressive properties (178) and also by healing damaged intestinal epithelium (179); however, in the case of antigen-mismatched bone marrow transplantation, donor MSCs can induce chronic GvHD by interacting with residual host T cells. Prochymal was first approved by the Food and Drug Administration against GvHD and is composed of BM-MSCs (180); it is mostly used in patients non-responsive to steroids and other immunosuppressive agents (181). Another drug based on MSCs—Alofisel—is based on expanded adipose-derived stem cells (182) and has been approved by the European Medicines Agency as therapy for complex perianal fistulas. Both of these drugs are allogeneic and derived from healthy adult individuals (183) (**Table 2**).

**Table 2:** Globally approved MSC products (104, 105).

Product name	Indication	Country	MSC type
Queencell (Anterogen Co., Ltd.)	Subcutaneous tissue defects	South Korea (2010)	Autologous AT-MSC
Cellgram-AMI (Pharmicell Co., Ltd.)	Acute myocardial infarction	South Korea (2011)	Autologous human BM-MSC
Cartistem (Medipost Co. Ltd.)	Knee articular cartilage defects	South Korea (2012)	Allogeneic human UC-MSC
Cupistem (Anterogen Co., Ltd.)	Crohn’s fistula	South Korea (2012)	Autologous human AT-MSC
Prochymal (Osiris Therapeutics Inc.) Remestemcel-L (Mesoblas Ltd.)	GvHD	Canada (2012) New Zealand (2012)	Allogeneic human BM-MSC
NeuroNata-R (Corestem Inc.)	Amyotrophic lateral sclerosis	South Korea (2014)	Autologous human BM-MSC
Temcell HS (JCR Pharmaceuticals)	GvHD	Japan (2015)	Allogeneic human BM-MSC
Stempeucel (Stempeutics Research PVT)	Critical limb ischemia	India (2016)	Allogeneic human BM-MSC
Stemirac (Nipro Corp.)	Spinal cord injury	Japan (2018)	Autologous human BM-MSC
Alofisel (TiGenix NV/Takeda)	Complex perinatal fistulas in Crohn’s disease	Europe (2018)	Allogeneic human AT-MSC

The regenerative potential of MSCs is still intensively explored. There is a wide MSC secretome spectrum to explore in order to broaden its application in regenerative medicine. There are many challenges and translational considerations in the clinical usage of MSCs, which include the following: MSC donor (age, gender, health status), tissue source (umbilical cord, adipose tissue, bone marrow), MSC manufacturing variables (isolation and expansion methods, culture conditions, cryopreservation, banking approach), MSC administration (patient population, route of delivery, dosage, frequency of dosage, dosing interval, using of biomaterials), and recipients (patient variability, disease severity, immune factors, host cytotoxicity, and responses). All the above variables need to be considered in the context of cell-based therapy applications in the clinic.

## 9 Discussion

A broad spectrum of diseases is related to an amplified immune response. The immune system is complex and multifactorial, providing footholds for possible immunomodulatory intervention. In this review, we outlined immunomodulators, such as the widely used corticosteroids, non-steroidal anti-inflammatory drugs, histamine antagonists, cellular signaling inhibitors, monoclonal antibodies, and interferons, and also explored the immunomodulatory properties of hMSCs or novel strategies with great potential to be implemented as future immune-related disease therapeutics, i.e., genomic strategies in immunomodulation (CRISPR–Cas9 and RNA interference).

The classic pharmacological approach, using drug classes such as corticosteroids, NSAIDs, and histamine antagonists, is the oldest known immunomodulatory intervention from all those described in our review, and over the years, these drugs have proven successful in the suppression of inflammatory processes in patients. However, it does not mean that are the best established and best known, since their action is not tissue- or cell-selective and thus can result in a plethora of side effects, especially after their long-term usage. The design of new compounds from these drug classes is currently aimed mostly at the improvement of their selectivity as well as pharmacodynamic and pharmacokinetic properties in comparison with currently used molecules. An interesting branch of small molecule immunomodulatory drugs is compounds that act on intracellular pathways, as inhibitors of selected protein kinases, thus allowing a more specific and targeted therapeutic approach.

Even greater selectivity toward targeting particular cellular and molecular components of the immune system has been achieved with antibody therapies and the introduction of mAb-based drugs in the 1990s, which are used in patients suffering from autoimmune diseases, such as Crohn’s disease, rheumatoid arthritis, lupus, psoriasis, and MS (110). Currently, the greatest challenge in mAb therapeutics is mainly their immunogenicity and the formation of antidrug antibodies which decrease their clinical efficacy; however, more refined methods of mAb production and the development of bispecific mAbs, which would also influence endogenous B-cell action (112), appear to be promising strategies of improving their action. It is, however, also worth pointing out that the therapeutic potential of mAbs is limited to extracellular targets, such as cytokines or receptors and other transmembrane proteins, and thus, they cannot directly target intracellular proteins.

Genomic strategies, which include RNAi using siRNAs or miRNAs and CRISPR–Cas9 on the other hand, allow for the selective targeting of every cellular protein, including transcription factors. Additionally, CRISPR–Cas9 gene editing also enables a stable replacement of particular DNA sequences within the genome. The modification of T cells by CRISPR–Cas9 in cancer has refreshed and augmented our existing therapeutic strategies (184). In 2018, the US Food and Drug Administration approved the first therapeutic agent which implements the mechanism of RNAi. However, in the case of both interventions, there are some heavy limitations, which need to be challenged, before they could be widely used in the treatment of immune-related disorders, such as their effective and specific transfer to a selected cell type in a complex eukaryotic organism and the prevention of off-target effects. On the other hand, the CRISPR–Cas9 system may offer a significant advantage also in basic preclinical research, aimed at expanding our knowledge about different autoimmune diseases, since it constitutes a convenient tool for identifying new targets related to their pathogenesis (185).

Last but not least, hMSCs constitute a promising tool in the modulation of the immune status of injured tissue. The undisputed advantage of MSCs is their low immunogenicity: MSCs constitute an inexhaustible source of the immunomodulatory secretome. Researchers and clinicians, however, need to face many challenges to unify outcomes arising from both basal research and clinical trials in terms of the gender, age, and health of the MSC donor; tissue source; MSC manufacturing conditions; MSC route of delivery; dosage and pattern of administration; and recipients (patient variability, disease severity, immune factors, host cytotoxicity, and responses) (186). All the above variables need to be considered in the context of cell-based therapy application in the clinic.

## 10 Concluding remarks

Immunomodulation is a challenging branch of medical science, and with the steady improvements in drug design, immunomodulators have become more selective and attenuate the side effects of novel pharmacological treatments (7–9). There are limitations in improving manufacturing capabilities: chemical formulation and delivery mechanisms of recently designed highly selective molecules to be safer and more efficacious therapeutic compounds (14, 18, 19). As in the case of treating diseases in general, these substantive advances need to be combined with a more judicious selection of disease indications and better-validated intervention pathways (148). In summary, introducing new therapeutic approaches to treating inflammatory and autoimmune diseases requires ongoing collaboration between clinics and basic research to better understand the complex interactions between individual components of the immune system to identify potentially new targets for more specific therapeutic interventions.

## Author contributions

MSt and MM: conceptualization and design. MSt, JD, and MM: data collection and analysis. MSt, JD, PM, and MSo: preparation of the original draft. MSt, JD, PM, MSo, and MM: revision and approval of the subsequent drafts of the manuscript. All authors contributed to the article and approved the submitted version.
